# Research on the Machinability of Micro-Tapered Hole Group in Piezoelectric Atomizer and the Improvement Method

**DOI:** 10.3390/s22207891

**Published:** 2022-10-17

**Authors:** Fan Zhang, Xi Huang, Bochuan Chen, Yuxuan Huo, Zheng Liu, Weirong Zhang, Mingdong Ma, Xiaosi Zhou, Zhongwei Liang, Zhenzhen Gui, Jianhui Zhang

**Affiliations:** School of Mechanical and Electrical Engineering, Guangzhou University, Guangzhou 510006, China

**Keywords:** piezoelectric atomizer, atomizing sheet, micro-tapered hole, laser drilling

## Abstract

A metal atomizing sheet with a group of micro-tapered holes is the core constituent of a piezoelectric atomizer. However, the diameters of large-end and small-end micro-tapered holes in industrial applications deviate from the design values by 15.25% and 15.83%, respectively, which adversely impacts the effect of atomizers. In this study, two main factors that influence the machining quality of tapered holes, the external vibration disturbance and the internal system errors inside the laser processor, were explored; consequently, the vibration model of the machining device and the laser drilling model were established, respectively. Based on the models and the experimental results, it was found that the errors in diameter caused by these two factors accounted for 20% and 67.87% of the total deviation, respectively. Finally, an improved method was proposed, where a damping system was added to the machining device, and the diameter of the initial laser spot was corrected. The measurement results of tapered holes machined by the improved method showed that the deviation of the large diameters and the small diameters from the design values declined to 4.85% and 4.83%, respectively. This study lays a foundation for the high-precision and large-scale industry of atomizing sheets, and provides a new research direction for enhancing the performance of atomizers.

## 1. Introduction

The piezoelectric atomizers that have emerged in recent years have the advantages of minimal noise, small atomizing quantities and small droplet size over traditional pneumatic atomizers; thus, they have been extensively applied in various fields such as ink-jet printing, spray cooling, biomedicine, spray machining, aromatherapy and odor reproduction [1,2,3,4,5,6,7]. Piezoelectric atomizers are divided into two structures, according to differences in the actuator: firstly, the liquid is atomized by high-frequency ultrasonic waves, as the actuator, generated due to the vibration of piezoelectric ceramics, and large droplets are filtered through small-holed metal meshes, thus completing atomization; this is referred to as a piezoelectric ultrasonic atomizer [8,9,10]. Secondly, small-holed metal meshes serve as the actuator under synchronous vibration with piezoelectric ceramics, thus completing atomization; this is called a piezoelectric mesh atomizer [11,12,13]. Therein, small-holed metal sheets are the core components of the two piezoelectric atomizer types, and these sheets have a direct bearing on the atomization effect.

Piezoelectric atomizers have been initially applied to the design of ink jet printers, in which wire meshes are used as metal atomizing sheets [14,15,16,17]. Since the size of atomized particles is directly influenced by the hole size on meshes, micro-holes with a smaller diameter are machined on metal sheets in order to acquire micro-ink droplets [18,19]. Afterwards, small tapered holes are machined by researchers on metal sheets to replace the original straight holes, and the capillary force of tapered holes can be utilized to promote liquid fracture and accelerate its jet velocity [20,21,22], thus further extending the application of piezoelectric atomizers in fields such as spray cooling, cell manipulation and high-viscosity liquid atomization [23,24,25,26,27].

Currently, piezoelectric atomizers have been applied to the field of inhalation drug delivery. Specifically, drugs directly act, through atomization, upon the upper respiratory tract and lungs, which substantially reduces the dosage and the damage to organisms. They are the best choice for treating COVID-19, asthma, pulmonary heart diseases, emphysema and pneumonia [28,29,30,31]. During the process of inhalation drug delivery, the depth of drug action is proportional to the size of the atomized particles; namely, the smaller the particle size, the deeper the drug action. Therefore, the size of atomized particles is required to be below 5 μm in order to truly realize pulmonary drug delivery [32].

The size of atomized particles has been reduced in the existing studies, mostly on the basis of two aspects: piezoelectric excitation parameters [33,34,35,36,37,38,39] and atomizers’ structural parameters [40,41,42,43,44,45]. However, atomizing sheets with tapered holes, which are the core components, have rarely been the research object, nor have they been explored in terms of the effects of machining technology and machining accuracy of micro-tapered holes on their atomization performance. In this study, the external vibration disturbance and the internal system errors inside the laser processor, both of which influence the machining quality of tapered holes, were explored. The vibration model of the machining device and the laser machining model are established methods. Through modeling and experimentation, the influence of these two factors on machining deviations of holes was quantitatively analyzed, and an improved method for machining equipment, in terms of structure and algorithm, was proposed. This study lays a foundation for the high-precision and large-scale industry of atomizing sheets, and provides a new research direction for enhancing the performance of atomizers.

## 2. Atomizer Structure and Laser Drilling Principle

### 2.1. Atomizer Structure

**Figure 1:**
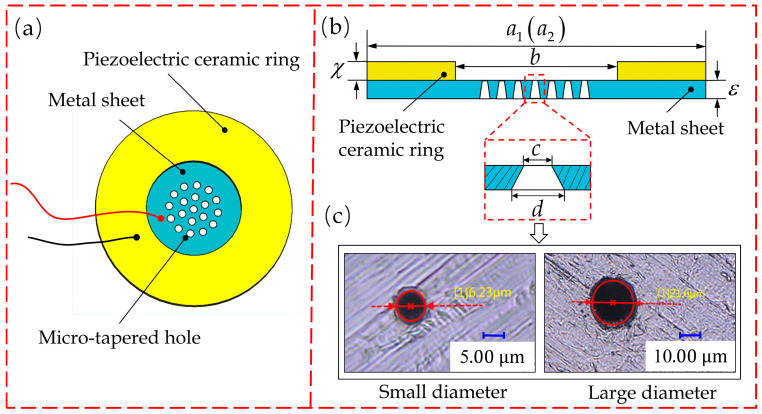
Structural drawing of atomizing sheet: (**a**) vertical view; (**b**) side view; (**c**) microscopical photographs of the small end and large end of a micro-tapered hole.

The atomizing sheet in a piezoelectric atomizer is formed by bonding a piezoelectric ceramic ring and a metal sheet with a group of micro-tapered holes (Figure 1). Figure 1a is a vertical view and Figure 1b presents a side view. A total of 300 micron-level tapered holes are machined on atomizing sheets that are currently used in industry, with their ultra-depth-of-field microphotographs shown in Figure 1c. In this study, the thickness, outer diameter and inner diameter of the used piezoelectric ceramics are denoted by χ, $a_{1}$ and b, respectively. The thickness and diameter of the metal sheet are expressed as ε and $a_{2}$, respectively (see details in Table 1).

### 2.2. Laser Drilling Principle

Currently, micro-tapered holes are machined mainly through micro-electric charge machining (EDM) [46,47], micro-electrochemical machining (ECM) [48,49], mini-type ultrasonic machining [50], laser processing technology [51] and other hybrid techniques [52,53]. The micro-tapered hole machining method that was explored in this study is the laser drilling technology which is widely applied in the industry; it belongs to a laser ablation technology (also referred to as evaporation process) [54]. This laser drilling technology is divided into two types: hot working and cold working, where the former means heating the surface of work piece materials through IR laser so that the materials are evaporated or vaporized to reach the goal of removing work piece materials; the latter refers to molecular bond rupture of work piece materials via UV lasers to reach the machining goal [55]. In general, hot working is mainly adopted to process holes with diameters of less than 5 μm; the diameter of micro-holes that are processed through cold working can reach a micron level, but with a very small taper angle and a high machining cost. Hence, the laser processing method of metal sheets probed in this study is the IR laser processing method that is commonly used in the industry at present.

**Figure 2:**
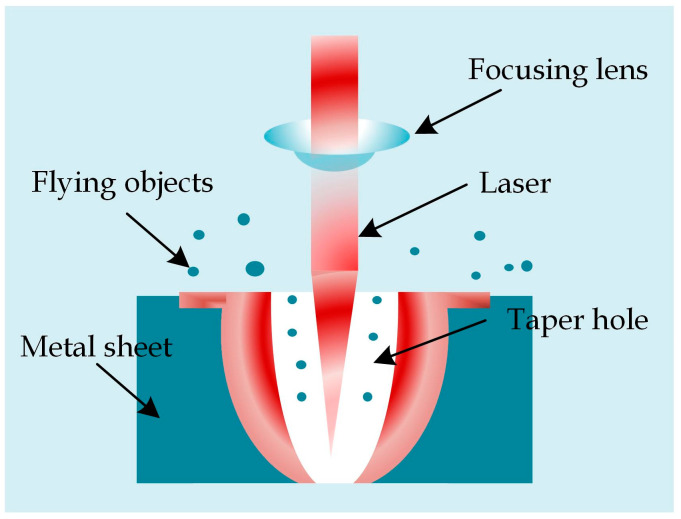
Schematic diagram of laser drilling.

The schematic diagram of IR laser-based metal sheet drilling is displayed in Figure 2. The laser beam that is emitted by the laser is incident onto a focusing lens through a light path, and finally the laser beam is focused onto the metal sheet to be worked, thus forming a light spot.

With the accumulation of time, energy is continuously accumulated at the light spot, which leads to an increase in temperature to a point that is slightly lower than the material melting point; then, laser-induced material damage initiates. In this case, the intense phase change of solid metal is the main feature, and the liquid phase appears firstly, followed by the gaseous phase [56]. As the heat energy continuously increases, the temperature also rises continuously, the thermal movement of gas molecules intensifies, their average velocity accelerates, and there are increasing molecules impacting on the plane within unit time, thus resulting in high pressure. The metal vapor is sprayed out from the bottom of the liquid phase under the high pressure, along with splashing, thus completing the laser drilling process.

**Figure 3:**
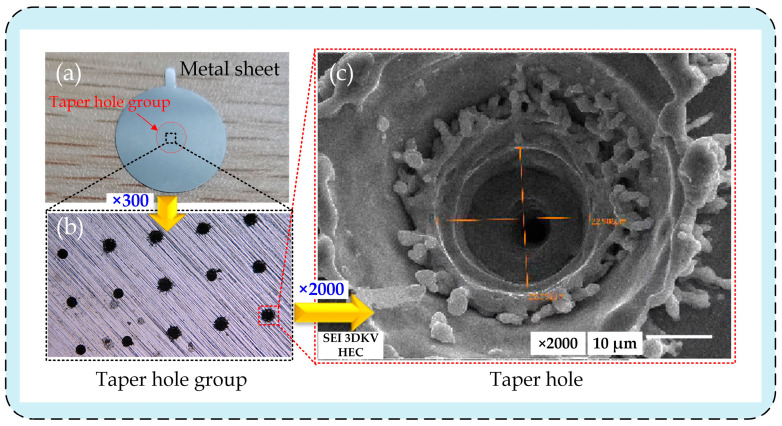
Tapered hole images at different magnifications: (**a**) metal sheet; (**b**) taper hole group; (**c**) taper hole.

The pictures of tapered holes on the metal sheet shot at different magnifications are exhibited in Figure 3. Figure 3a displays the image of a metal sheet that was taken by a single lens reflex (SLR) camera; a red-colored circle indicates a tapered hole group, which can be observed in the center of the metal sheet. Figure 3b presents the image of the tapered hole group on the metal sheet taken by an ultra-depth-of-field microscope (VH-20, Keyence, Osaka, Japan) at a magnification of 300×. The 2000× image is shown in Figure 3c, from which it can be seen that the inner wall of micro-tapered holes is not smooth, but presents an obvious three-layer stepped structure, which is attributed to the machining procedures used to make micro-tapered holes. In contrast to machining straight holes, the machining requirements of micro-tapered holes at different taper angles can be realized only by repeatedly adjusting the spot diameter. An image showing a micro-tapered hole with three changes in diameter is displayed in Figure 3c.

## 3. Theoretical Analysis

### 3.1. Establishment of Vibration Model

**Figure 4:**
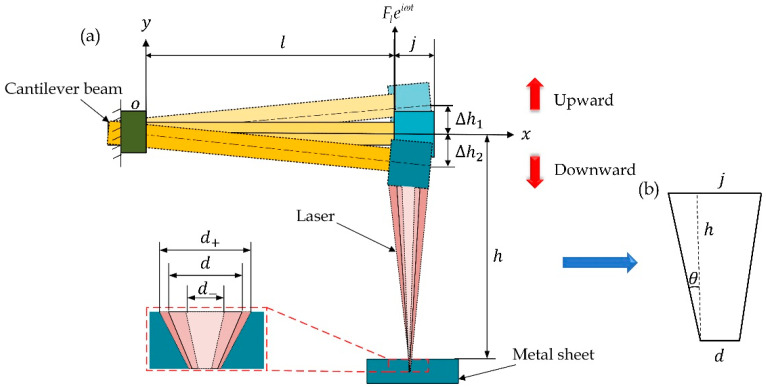
Coordinates of laser machine and vibration model: (**a**) schematic diagram of influence of laser machine vibration on spot size; (**b**) schematic diagram of the relationship between the geometric dimensions, optical path and light spot of the laser processor.

The laser machine-based drilling of metal sheets is influenced by the external environment and generates vibration, which affects the laser machining accuracy of metal sheets. Therefore, the structure of the laser machine was simplified into a cantilever structure (Figure 4). The laser machine vibrates up and down when affected. Therein, the light color shade represents the state of the laser machine vibrating upward to the highest position, the medium color shade stands for its state when vibrating to the horizontal position, and the dark color shade denotes its state when vibrating downward to the lowest position. Figure 4b displays the laser-size relational graph, where θ is the included angle between the laser and the externally added micro-lens perpendicular to the laser, j represents the length of the added micro-lens, and h denotes the distance from the laser machine to the metal sheet. d, $d_{+}$ and $d_{-}$ are the diameters of tapered holes on the metal sheet under the ideal state, namely no vibration of the laser machine, in case of downward vibration of the laser machine under working conditions, and in the event of upward vibration of the laser machine under working conditions, respectively. $\Delta h_{1}$ and $\Delta h_{2}$ stand for the displacement when the laser machine vibrates upward and downward, respectively, and l is the arm length of the laser machine.

Based on the theoretical knowledge of Euler–Bernoulli beam equation, the vibration differential equation for the free vibration of beams was acquired, as shown below:

(1) $$m\left(x\right)\frac{\partial^{2}u\left(x,t\right)}{\partial t^{2}}+EI\left(x\right)\frac{\partial^{4}u\left(x,t\right)}{\partial x^{4}}=0$$

where EI(x), m(x) and u(x,t) represent the flexural rigidity, the mass per unit length of cantilever beam and its vibration displacement, respectively. With the method of separation of variables adopted, the solution was set into the following form:

(2) u(x,t)=ϕ(x)q(t)

The separation constant was set as $\omega^{2}$ by combining Equations (1) and (2). Accordingly, the ordinary differential motion equation in the spatial domain could be deduced as follows:

(3) $$\phi^{\left(4\right)}\left(x\right)-\alpha^{4}\phi \left(x\right)=0$$

where $\alpha_{n}=\sqrt[{4}]{\omega_{n}{}^{2}m/EI}$ is a constant, which is determined according to the initial conditions of the laser machine; $\omega_{n}$ represents the inherent vibration frequency.

The general solution could be expressed through the trigonometric function and hyperbolic function, as shown below:

(4) ϕ(x)=Asinαx+Bcosαx+Csinhαx+Dcoshαx

where A, B, C and D are constants, which are determined through boundary conditions.

The boundary conditions of the cantilever beam were set as follows: when x=0, ϕ(0)=0; when x=l, $\phi^{\prime }\left(l\right)=\phi^{\prime \prime }\left(l\right)=0$. The functional expression of the n(th)-order inherent vibration mode of the cantilever beam was solved as below:

(5) $$\phi_{n}\left(x\right)=\cosh\alpha_{n}x-\cos\alpha_{n}x-E_{n}\left(\sinh\alpha_{n}x-\sin\alpha_{n}x\right)$$

where $E_{n}=\frac{\sinh\alpha_{n}l-\sin\alpha_{n}l}{\cosh\alpha_{n}l+\cos\alpha_{n}l}$.

After Equation (5) was simplified, the n(th) order inherent vibration mode function of the cantilever beam was acquired, as follows:

(6) $$\phi_{n}\left(x\right)=\frac{2\sinh\alpha_{n}x\sin\alpha_{n}x}{\cosh\alpha_{n}x+\cos\alpha_{n}x}$$

When the exciting force $F_{l}e^{i\omega t}$ acts upon the cantilever beam, the micro-element dx is taken as the detached body; the effects of rotational inertia and shear deformation could be neglected; thus, the following forced vibration differential equation of the cantilever beam was acquired:

(7) $$\frac{d^{2}q_{n}\left(t\right)}{dt^{2}}+2\xi_{n}\omega_{n}\frac{dq_{n}\left(t\right)}{dt}+\omega_{n}^{2}q_{n}\left(t\right)=F_{n}e^{i\omega t}$$

where $q_{n}\left(t\right)$ denotes the forced response of the single degree-of-freedom (SDOF) system, $\xi_{n}$ is its relative damping coefficient, $\omega_{n}$ represents the inherent vibration frequency, and $F_{n}=F_{l}\phi \left(x\right)$ is the amplitude of the n(th)-order mode force.

According to the force analysis shown in Figure 4 above, the cantilever beam is subjected to the concentrated exciting force $F\left(t\right)=F_{l}e^{i\omega t}$ and x=l. The unit pulse function with the independent variable of (x−l) can be used to express the concentrated exciting force as a distributed exciting force:

(8) $$F\left(x,t\right)=F_{l}e^{i\omega t}\delta \left(x-l\right)$$

Based on the nature of the unit pulse function:

(9) $$\int_{-\infty }^{\infty }\delta \left(x-l\right)G\left(x\right)=G\left(l\right)$$

The *n*th-order mode force was obtained by combining Equations (8) and (9), as shown below:

(10) $$F_{n}\left(t\right)=\int_{0}^{l}F\left(t\right)\delta \left(x-l\right)\phi_{n}\left(x\right)dx=F_{l}e^{i\omega t}\phi_{n}\left(l\right)$$

where $\phi_{n}\left(x\right)$ is the *n*th-order vibration mode function of the cantilever beam, and l represents the total length of the cantilever beam of the laser machine.

With Equations (7) and (10) combined, the forced vibration differential equation under the concentrated exciting force was obtained:

(11) $$\frac{d^{2}q_{n}\left(t\right)}{dt^{2}}+2\xi_{n}\omega_{n}\frac{dq_{n}\left(t\right)}{dt}+\omega_{n}^{2}q_{n}\left(t\right)=F_{l}e^{i\omega t}\phi \left(l\right)$$

On this basis, the response of the cantilever beam under the concentrated exciting force could be acquired:

(12) $$u\left(x,t\right)=\sum_{n=1}^{\infty }\phi_{n}\left(x\right)q_{n}\left(t\right)=\sum_{n=1}^{\infty }\frac{e^{i\left(\omega t-\varphi_{n}\right)}F_{l}\phi_{n}\left(l\right)\phi_{n}\left(x\right)}{{\bar{\omega }}_{n}^{2}\sqrt{{\left(1-{\bar{\omega }}_{n}^{2}\right)}^{2}+{\left(2\xi_{n}{\bar{\omega }}_{n}\right)}^{2}}}$$

The following dimensionless parameters were introduced: frequency ratio (${\bar{\omega }}_{n}=\omega /\omega_{n}$), excitation frequency (ω), inherent frequency ($\omega_{n}$), and the *n*th-order vibration mode function of the cantilever beam [$\phi_{n}\left(x\right)$]. The expression of $\phi_{n}\left(x\right)$ is denoted by Equation (6).

The forced response at the end of the laser machine to the steady-state displacement under the action of the exciting force was acquired, as per Equation (12):

(13) $$u\left(l,t\right)=\sum_{n=1}^{\infty }\phi_{n}\left(l\right)q_{n}\left(t\right)=\sum_{n=1}^{\infty }\frac{e^{i\left(\omega t-\varphi_{n}\right)}F_{l}\phi_{n}^{2}\left(l\right)}{{\bar{\omega }}_{n}^{2}\sqrt{{\left(1-{\bar{\omega }}_{n}^{2}\right)}^{2}+{\left(2\xi_{n}{\bar{\omega }}_{n}\right)}^{2}}}$$

The laser machine was assumed to vibrate upward; thus, the distance from the laser machine to the metal sheet surface is as follows:

(14) $$h+\Delta h_{1}=h+\left|u\left(l,t\right)\right|$$

When the laser machine vibrates downward, the distance from the laser machine to the metal sheet surface is as shown below:

(15) $$h-\Delta h_{2}=h-\left|u\left(l,t\right)\right|$$

The vibration displacement of the laser machine under forced vibration is associated with laser drilling under working conditions. According to the vibration of the laser machine, the variable quantity of the diameter due to the vibration during laser drilling can be deducted. θ is the included angle between the laser and the micro-lens perpendicular to the laser, j represents the length of the externally added micro-lens, and h denotes the distance from the laser machine to the metal sheet. When the laser machine vibrates upward under the forced vibration of the laser machine, the diameter of tapered holes on the metal sheet is as follows:

(16) $$d_{-}=j-2\tan\theta \left[h+\left|\sum_{n=1}^{\infty }\frac{e^{i\left(\omega t_{-}-\varphi_{n}\right)}F_{l}\phi_{n}^{2}\left(l\right)}{{\bar{\omega }}_{n}^{2}\sqrt{{\left(1-{\bar{\omega }}_{n}^{2}\right)}^{2}+{\left(2\xi_{n}{\bar{\omega }}_{n}\right)}^{2}}}\right|\right]$$

If the laser machine vibrates downward under the forced vibration excitation, the diameter of tapered holes on the metal sheet is expressed as below:

(17) $$d_{+}=j-2\tan\theta \left[h-\left|\sum_{n=1}^{\infty }\frac{e^{i\left(\omega t_{+}-\varphi_{n}\right)}F_{l}\phi_{n}^{2}\left(l\right)}{{\bar{\omega }}_{n}^{2}\sqrt{{\left(1-{\bar{\omega }}_{n}^{2}\right)}^{2}+{\left(2\xi_{n}{\bar{\omega }}_{n}\right)}^{2}}}\right|\right]$$

where d, $d_{+}$ and $d_{-}$ represent the diameters of tapered holes on the metal sheet under the ideal state, namely no vibration of the laser machine, in the case of downward vibration under working conditions, and in the event of upward vibration under working conditions, respectively. u(l,t) is the vibration displacement generated at the end of the laser machine due to the external environment, in which l stands for the arm length of the laser machine.

### 3.2. Diameter Distribution Model of Tapered Holes on the Metal Sheet

In nature and production, some phenomena are influenced by many mutually dependent factors. If each factor exerts a very minor influence, the total influence can be considered to follow a normal distribution. The diameters of tapered holes on the metal sheet can be considered to present a normal distribution. In this experiment, the number of samples used to explore the diameter of the metal sheet was set as N, and the average value of the diameter among the experimental samples could be expressed, as shown below:

(18) $${\bar{X}}_{e}=\frac{1}{N}\sum_{i=1}^{N}X_{i}$$

The variance was denoted as shown below:

(19) $$\sigma_{e}^{2}=\frac{1}{N}{\sum_{i=1}^{N}\left(X_{i}-{\bar{X}}_{e}\right)}^{2}$$

The probability density function of the hole size distribution was determined as follows:

(20) $$\varphi \left(X\right)=\frac{1}{\sqrt{2\pi }\sigma }e^{-\frac{{\left(X-\bar{X}\right)}^{2}}{2\sigma^{2}}}$$

where $\bar{X}$ and σ stand for the average value and the standard deviation of the tapered hole diameter on the metal sheet, respectively.

In order to verify whether systematic error disturbance existed in the laser drilling process of the metal sheet, the data that were obtained on the basis of the hypothesis of normal distribution were comparatively analyzed with the actually measured data of hole sizes of the metal atomizing sheets. The average value and variance of the tapered hole diameter under the ideal machining state without external disturbance were set as ${\bar{X}}_{S}$ and $\sigma_{s}^{2}$, respectively, both of which depended on the optical and electronic control characteristics of the laser machine.

During the machining process, the average value and the variance value of the tapered hole diameter was required to meet the following conditions:

(21) $$\frac{\left|{\bar{X}}_{s}-{\bar{X}}_{e}\right|}{{\bar{X}}_{s}}\leq \beta$$

(22) $$\frac{\left|\sigma_{s}^{2}-\sigma_{e}^{2}\right|}{\sigma_{s}^{2}}\leq 0.9\beta$$

According to practical application in the atomization field, β was set as 0.05. If experimental results did not meet Equations (21) and (22), the tapered holes on the machined metal sheet were too large, thus failing the practical application in the atomization field. Given this, the follow-up experimental error analysis would be done. If the experimental results met Equations (21) and (22), the experimental errors fell into a permissible range, and thus, the tapered holes were applicable in the atomization field.

### 3.3. Laser Drilling Model under Three Working Procedures

**Figure 5:**
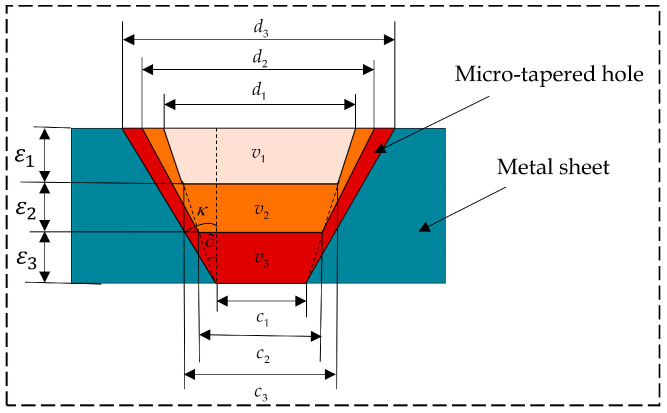
Schematic diagram of area occupied by three working procedures in the laser drilling process.

Since three working procedures are currently applied to tapered hole machining for most atomizing sheets in the industry, a laser drilling model that was specific to the three working procedures was established in this study; furthermore, the relationship between the laser spot diameter and the machined hole diameter under each working procedure was established. Figure 5 shows the schematic diagram of the tapered hole laser drilling model that was completed through three working procedures. 

Under the laser multi-pulse action in each working procedure, the materials in the depth direction were continuously melted and sprayed out, and the hole depth increased, accompanied by continuous melting at the edge of the molten pool through thermal diffusion. After the first procedure, the machined diameter exceeded the diameter of the laser spot, since the upper end of the metal sheet contacted the air with the presence of oxygen, resulting in melting that was more intense. Therefore, after the three procedures, the diameter expanded further to finish the process. In order to discuss the relationship between the machined diameter $d_{3}$ and the laser spot diameter $d_{1}$, a drilling machining model was established using an energy balanced model.

The model was divided into the main melted zone ($v_{1}$ in Figure 5) and the secondary melted zone ($v_{2}$ and $v_{3}$ in Figure 5). The molten diameters and depths through the three working procedures were calculated based on identical energy among the three working procedures. The model in this paper was for a cone angle κ that was 0.08°. Therefore, the process was fixed as 3. If the cone angle changed, the idea of building the model remained the same, but the number of processes in the model needed to be changed in order to obtain the appropriate model.

The energy required by laser drilling could be determined as follows:

(23) Q=ηE

where Q is the energy required by laser drilling, η=0.8, which is a loss coefficient; E=IST represents the pulse energy; I stands for the power density of laser drilling; S is the molten area of metal sheet drilling; and T denotes the pulse time required. Concrete parameter values are listed in the following Table 2.

The micro-holes in the metal sheet were tapered holes whose volume could be approximated as that of cones; thus, the volume could be expressed as follows:

(24) $$v=\frac{1}{12}\pi \varepsilon \left(d^{2}+c^{2}+dc\right)$$

where v is the molten volume of the metal sheet, ε represents the total molten depth of tapered holes on the metal sheet, and d and c stand for the large diameter and the small diameter in the melting process of the metal sheet, respectively.

Laser drilling experiences two basic processes: material melting and vaporization. The material is assumed to be completely vaporized under the laser action, and the material depth formed is expressed as shown below:

(25) $$\varepsilon =\frac{12Q}{\pi \left(d^{2}+c^{2}+dc\right)\rho \left[C\left(t-t_{0}\right)+L_{m}+L_{V}\right]}$$

where C stands for the specific heat capacity of the metal sheet, t is the temperature required for melting, ρ is the density of the metal sheet, and ε represents the total melting depth of tapered holes on the metal sheet. $L_{m}$ and $L_{v}$ denote the latent heat of melting and the latent heat of vaporization, respectively. The concrete parameter values are displayed in the following Table 2.

The correspondence among the three working procedures is exhibited as follows:

(26) $$\{\begin{array}{c} \varepsilon =\varepsilon_{1}+\varepsilon_{2}+\varepsilon_{3}\\ v_{1}\approx v_{2}\approx v_{3}\\ Q_{1}=Q_{2}=Q_{3} \end{array}$$

where $\varepsilon_{1}$, $\varepsilon_{2}$ and $\varepsilon_{3}$, represent the melting depths under the three working procedures, respectively; $v_{1}$, $v_{2}$ and $v_{3}$ are the melting volumes under the three working procedures, respectively; $Q_{1}$, $Q_{2}$ and $Q_{3}$ are the energies required by melting under the three working procedures, respectively.

Since the energy required was basically identical under the three working procedures, the melting depth under the three working procedures was obtained by combining Equation (25), as exhibited by the following matrix:

(27) $$\left[\begin{array}{c} \varepsilon_{1}\\ \varepsilon_{2}+\varepsilon_{1}\\ \varepsilon_{3}+\varepsilon_{2}+\varepsilon_{1} \end{array}\right]=\left[\begin{array}{c} {\left(d_{1}^{2}+c_{1}^{2}+d_{1}c_{1}\right)}^{-1}\\ 2{\left(d_{2}^{2}+c_{2}^{2}+d_{2}c_{2}\right)}^{-1}\\ 3{\left(d_{3}^{2}+c_{3}^{2}+d_{3}c_{3}\right)}^{-1} \end{array}\right]\frac{12Q}{\pi \rho \left[C\left(t-t_{0}\right)+L_{m}+L_{v}\right]}$$

where $d_{1}$ and $c_{3}$ were determined through experimentation.

According to the triangular relationship, the taper angle of tapered holes on the metal sheet could be acquired as follows:

(28) $$\partial =\arctan\frac{d_{1}-c_{3}}{4\varepsilon }$$

According to Equation (28), the equivalent relationship between the small diameter of the metal sheet under the following working procedure and that under the second working procedure could be expressed by the following matrix:

(29) $$\left[\begin{array}{c} c_{1}\\ c_{2}\\ c_{3} \end{array}\right]=\left[\begin{array}{c} d_{1}\\ d_{1}\\ d_{1} \end{array}\right]-\left[\begin{array}{c} \varepsilon_{1}\\ \varepsilon_{1}+\varepsilon_{2}\\ \varepsilon_{1}+\varepsilon_{2}+\varepsilon_{3} \end{array}\right]{\left(4\tan\partial \right)}^{-1}$$

where ∂ is the included angle between large and small holes on the metal sheet in the main molten zone. The diameter of the metal sheet after the three working procedures was solved through Equations (27)–(29).

(30) $$d_{3}{}^{2}=\frac{36Q}{\pi \rho \left[C\left(t-t_{0}\right)+L_{m}+L_{v}\right]\varepsilon }-4{\left(\frac{d_{1}}{2}-\frac{\varepsilon }{2\tan\partial }\right)}^{2}-2d_{3}\left(\frac{d_{1}}{2}-\frac{\varepsilon }{2\tan\partial }\right)$$

## 4. Measurement of Tapered Hole Diameter on the Metal Sheet

Micro-tapered holes are significant constituents of atomizing sheets, which implement atomization through the pumping effect of micro-tapered holes. Hence, the size of micro-tapered holes and their diameter distribution exert important effects on atomization. In this study, the tapered hole diameters of the metal sheet were measured and analyzed, in order to judge whether the tapered holes of the metal sheet, which machined through the current process, met the requirements of Equations (21) and (22), namely to control the deviation of the average value to within 0.05 and the variance value to within 0.45.

**Figure 6:**
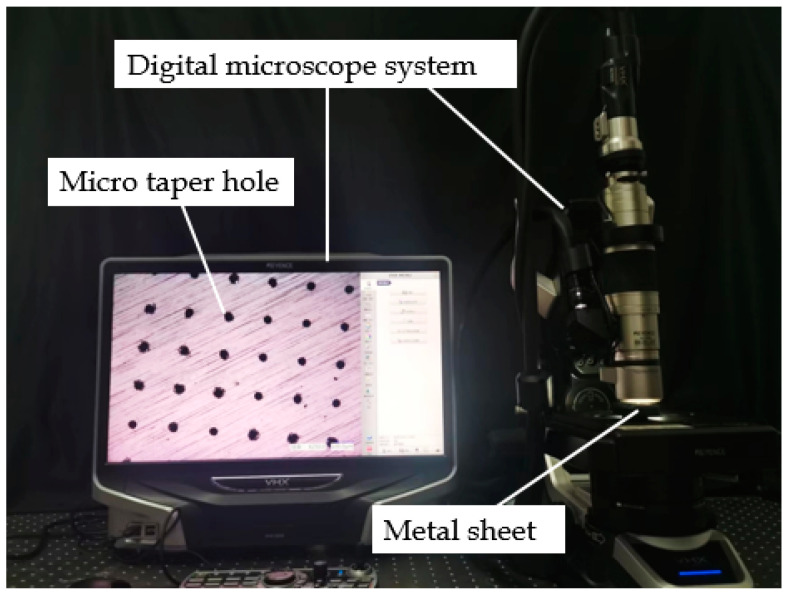
A photograph of the measurement setup of the micropore diameter.

Tapered hole diameters of the metal sheet were determined using an ultra-depth-of-field 3D microscope system (VHX-600, Keyence, Osaka, Japan), and a microscope system focusing lens (VH-20, Keyence, Osaka, Japan), as shown in Figure 6. A total of five metal sheets were randomly selected in this experiment; two holes were randomly selected in the central, upper, lower, left and right zones of the metal sheet, and a total of 50 measured large diameters and small diameters were obtained.

**Figure 7:**
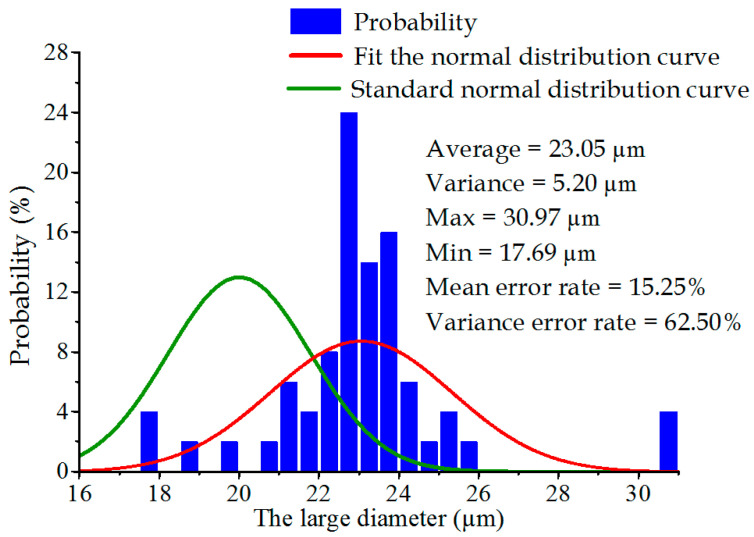
Distribution of the large diameter.

**Figure 8:**
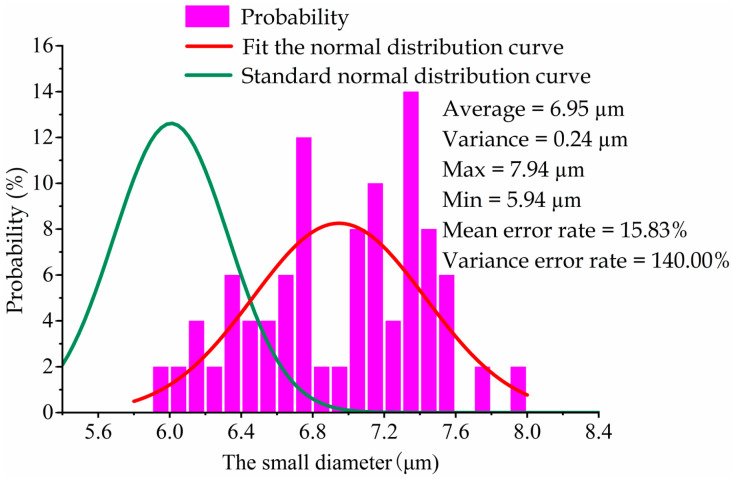
Distribution of the small diameter.

In this experiment, the large diameter of each metal sheet was measured under 1000× focusing conditions with the ultra-depth-of-field microscope, and the small diameter was measured under 2000× focusing conditions. During the laser drilling process, the large diameter ${\bar{X}}_{Sl}$ and small diameter ${\bar{X}}_{ss}$ of the laser machine were set as 20 μm and 6 μm, respectively. The nominal machining variance of this device was 3.2 μm for the large diameter and 0.10 μm for the small diameter. According to Equation (20), the theoretical diameter distribution curve of this laser machine could be obtained, which served as the machined standard value in this study. In Figure 7 and Figure 8, the green curve denotes the machined standard values of the large diameter and the small diameter, respectively; the red and purplish red histograms represent the experimentally measured data of the large diameter and the small diameter, respectively; and the red curve represents the fitted normal distribution curve.

The average values of the large diameter and the small diameter were experimentally calculated as 23.05 μm and 6.95 μm, respectively, through Equation (18). According to Equation (19), the two variances of the large diameter and the small diameter were solved as 5.2 μm and 0.24 μm, respectively. As per Equations (21) and (22), the errors of the average values for the large diameter and the small diameter were calculated as 15.25% and 15.83%, respectively, and the errors of the variance values for the large diameter and the small diameter were calculated as 62.50% and 140.00%, respectively; these were both much greater than the critical value, thus failing to meet the practical application in the atomization field.

## 5. Experimental Analysis of Error Interference

The above errors may have derived from external noise and the processing algorithm of the laser system. In this section, the error sources were determined through two experimental parts. First, the vibration noise in the external environment was measured, followed by the pit experiment of single process to analyze the system errors in the laser machine.

### 5.1. Experiment on Vibration Interference Errors in the External Environment

**Figure 9:**
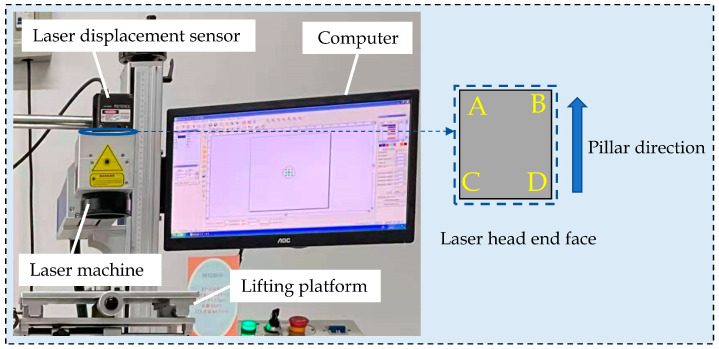
Physical drawing of vibration displacement test in the external environment.

The experimental device used for external environmental vibration is displayed in Figure 9. Therein, A, B, C and D are located on the upper surface of the laser head in the laser machine, C and D are at the end of the laser head, and A and B are closer to the pillar end than C and D. In the experiment, the vibration displacements at four end points on the upper surface of the laser machine under non-working conditions were measured using a laser displacement sensor, in order to analyze the effect of external environmental vibration on the laser machine.

**Figure 10:**
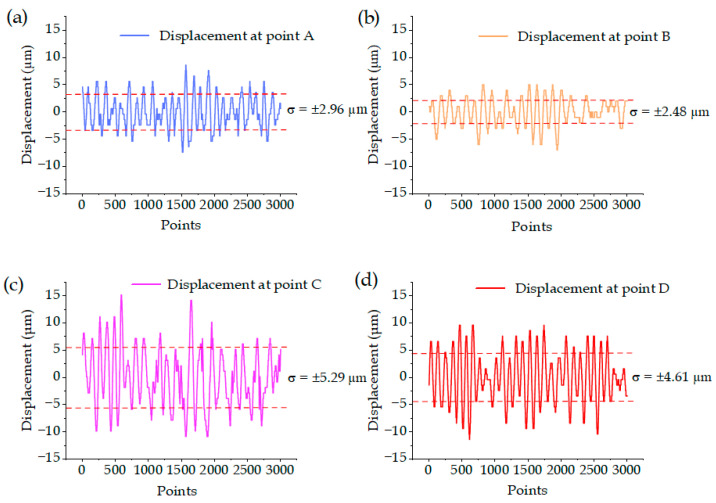
Point displacement vibration diagram of the laser machine: (**a**) vibration displacement diagram of point A; (**b**) vibration displacement diagram of point B; (**c**) vibration displacement diagram of point C; (**d**) vibration displacement diagram of point D.

According to Equation (12), the vibration displacements at points A and B of the laser machine were smaller than those at points C and D, due to differences in the arm length. The vibration displacements at the four points are exhibited in Figure 10. For the experimental results, upward vibration was set as positive vibration, and downward vibration was set as negative vibration. It could be known from experimental calculation results that the error intervals of the vibration displacements at points A, B, C and D were ±2.96 μm, ±2.48 μm, ±5.29 μm and ±4.61 μm, respectively, indicating that the error interval at points C and D were larger than those at points A and B. The larger error interval denoted greater data fluctuation and greater vibration displacement; thus, the theoretical values were consistent with the experimental data. With the vibration displacements at four points combined, the average value of the upward vibration displacement of the laser machine under the action of the external environment was 9.60 μm, and that of the downward vibration displacement was 9.15 μm. As a result, the large diameter increased by 0.61 μm, which accounted for 20.00% of the error, as seen in Equation (17).

**Figure 11:**
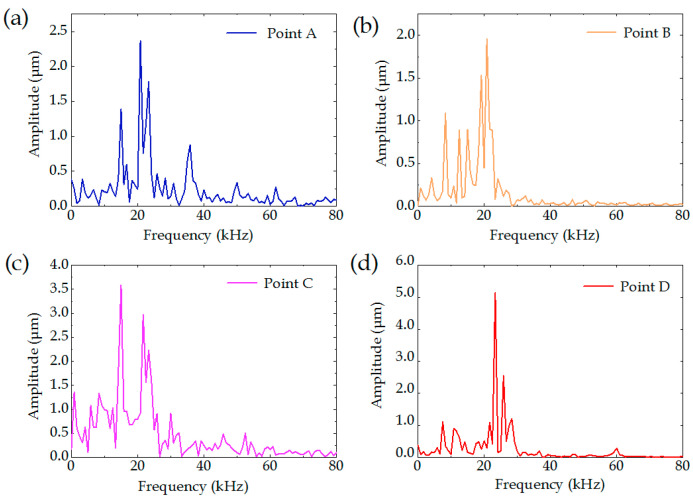
Amplitude–frequency diagram of vibrations on the laser machine: (**a**) amplitude–frequency diagram of point A; (**b**) amplitude–frequency diagram of point B; (**c**) amplitude–frequency diagram of point C; (**d**) amplitude–frequency diagram of point D.

The Fourier transform converts the time domain to the frequency domain. The MATLAB program was used to analyze the above experimental data in the frequency domain. The above experimental data were subjected to the frequency-domain analysis, as shown in Figure 11. At all four points, a peak value of the vibration amplitude appeared near 22 kHz, indicating that the plant of the laser machine was influenced by other nearby machines and machine tools, and that forced vibration at a frequency of 22 kHz was generated to the cantilever beam of the machine, thus influencing the machining quality of the tapered holes.

### 5.2. Interference Error Experiment on the Electrical System and Optical Control System Inside the Laser Machine

In order to eliminate the interference error that was imposed by external vibration on the laser machine, an experiment was carried out when other machines were halted at midnight to test whether errors existed in the laser machine’s control system. In the machining process of tapered holes, the machining of one hole was completed generally through three working procedures. The edge of the hole molten pool was continuously melted due to heat dissipation between working procedures. In this experiment, only one working procedure was completed through program setting, in order to machine a group of pits whose diameter was measured and considered to be the initial laser spot diameter of the first procedure, shown as $d_{1}$ in Figure 5. Moreover, the system errors inside the laser machine were investigated by analyzing the diameter distribution in the pit.

**Figure 12:**
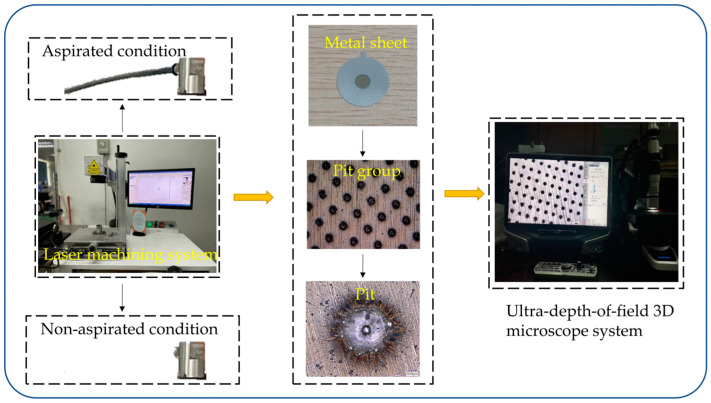
Experimental drawing of pit drilling and measurement.

The experimental device is exhibited in Figure 12. A group of pits were machined on 20 metal sheets, where 5 sheets were randomly selected for pit diameter measurement. For one sheet, two pits were randomly selected from the central, upper, lower, left and right zones of the metal sheet for measurement; thus, 50 measured pit diameter data were obtained in total. The pit diameter is the parameter $d_{1}$ in the first working procedure, and the hole diameter after the three working procedures could be subsequently calculated through pit parameters using Equation (30). Pits were drilled in the metal sheet under the aspirated state and the non-aspirated state. A sucking-off plant was added under the aspirated state relative to the non-aspirated state, in order to adsorb the gas and remove liquid molten slags that were generated during the laser drilling process; then, the inner wall of the hole became smoother, and burrs were reduced, so as to improve hole quality.

**Figure 13:**
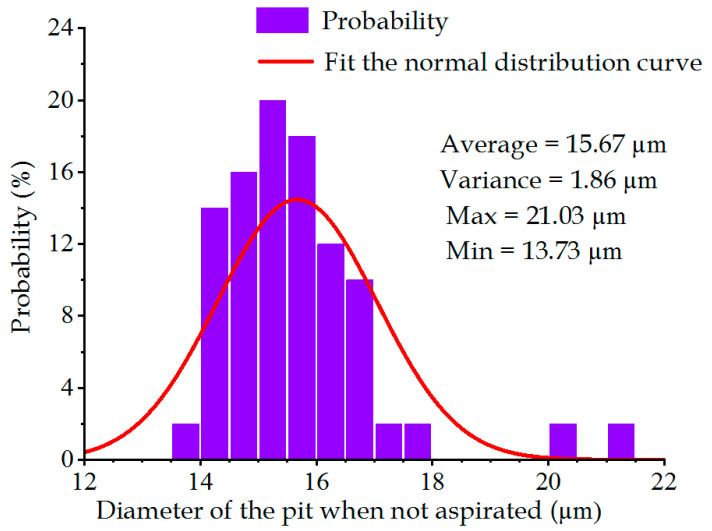
Distribution of pit diameters under the non-aspirated state.

**Figure 14:**
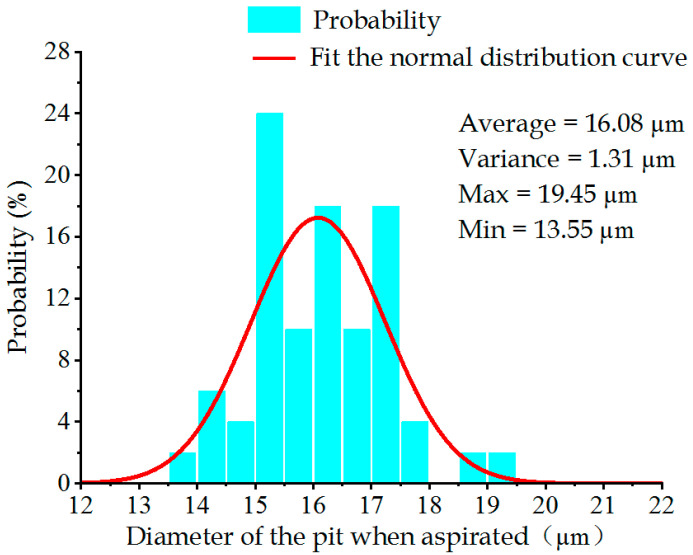
Distribution of pit diameters under the aspirated state.

Figure 13 and Figure 14 display the pit diameter–frequency relational graphs of the metal sheet under the aspirated and non-aspirated states. Under the non-aspirated state, the average value and variance of the pit diameter were 15.67 μm and 1.86 μm, respectively, which became 16.08 μm and 1.31 μm under the aspirated state, respectively. It could be observed that under the aspirated state, the average value of pit diameter was greater than that under the non-aspirated state. The reason for this is because as air was sucked off under the aspirated state of the metal sheet, it was subjected to upward and downward vibration, and deviations were generated. In order to control single variables, therefore, the pit diameter under the non-aspirated state was taken for the analytical calculation.

1. The pit diameter under the non-aspirated state was analytically calculated. The average value $d_{1}$ of the pit diameter was known as 15.67 μm. According to Equations (27)–(30), the depth $\varepsilon_{1}$ of the first working procedure was 18 μm. Given the identical volume of the metal sheet molten by laser energy each time, the depth $\varepsilon_{2}$ after the second working procedure was about 17 μm, the pit diameter $d_{2}$ was about 18.87 μm, the depth $\varepsilon_{3}$ after the third working procedure was 15 μm, and the pit diameter $d_{3}$ was about 22.07 μm. Since the preset value of the large diameter was 20 μm, the hole diameter of the metal sheet increased by 2.07 μm, owing the error in the processing algorithm. Therefore, the initial laser spot diameter was set to be too large due to the machining algorithm.
2. The laser machine consists of five major parts: a solid-state laser, electrical system, optical system, and a three-coordinate moving table. In the laser machine, the optical system and control system influence the laser beam to accurately focus on the machined part of the work piece, including the laser focusing device and the observation and aiming device. Laser focusing deviations led to the enlargement of hole diameter, due to the internal error of the device, the vibration of the metal sheet and its uneven surface. The laser beam energy supplied by the electrical system to the laser was inconsistent; thus, so was the laser energy between pits, accompanied by the unconcentrated diameter distribution.

### 5.3. Error Analysis

In summary of the above experiment, the large diameter of the metal sheet was analyzed. According to the overall error analysis of the large hole diameter, the average value of the large hole diameter on the metal sheet was 23.05 μm in the experiment, and that under the standard state it was 20 μm, with a difference value of 3.05 μm. This resulted from the following reasons:

- The average value of downward vibration displacement of the laser machine was 9.15 μm; namely, the distance from the laser machine to the metal sheet was shortened to 9.15 μm. As a result, the large diameter increased by 0.61 μm, which accounted for 20.00% of the error as seen in Equation (17).
- The tapered holes of the metal sheet were subjected to outward excessive melting in the machining process due to the initial spot diameter that was set too large. Through the processing algorithm Equation (30), the large diameter after the three working procedures was calculated to be 22.07 μm; thus, the large diameter increased by 2.07 μm, accounting for 67.87% of the error.
- Due to the optical system and the control system of the laser machine, the laser could not be accurately focused on the work piece, thus leading to positional deviation and error in the laser energy supplied by the electrical system to the laser; moreover, the metal sheet was uneven as a result of the suction device. All of these factors led to the inconsistent laser energy between holes, and this deviation accounted for 12.13% of the error.

## 6. Error Correction

### 6.1. Correction of Interference Error Induced by External Environmental Vibration

**Figure 15:**
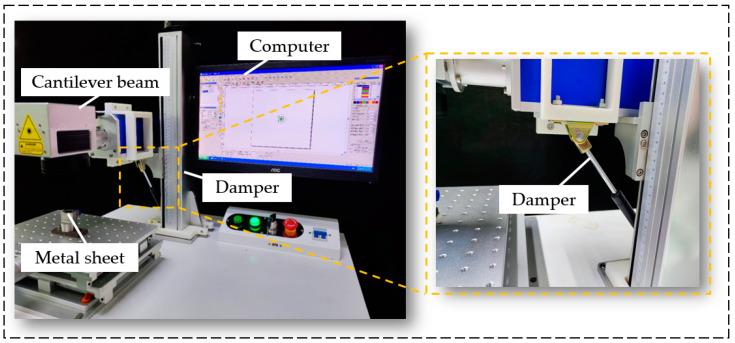
Schematic diagram of installing a damper on the laser machine.

The laser drilling process is influenced by an externally noisy environment. In this study, the structure of the laser machine was improved, thus reducing vibrations of the laser machine under working conditions. Specifically, as shown in Figure 15, a damper was added to the laser machine to relieve the vibration interference brought by the external environment.

### 6.2. Error Correction between Electrical System and Optical Control System Inside the Laser Machine

- Since the machined diameter exceeded the standard value due to the inappropriate setting of the initial diameter of the laser spot, according to Equation (30), it could be inferred that $d_{1}$ should be 14.72 μm when $d_{3}$ is 20 μm. Diameter $d_{1}$ was changed by reducing the laser energy to 1.75$\times 10^{-4}$ J.
- The error induced by the optical system and the electrical system in the laser machine could be corrected as follows: firstly, for the focusing deviation, the offset compensation could be set to ensure machining positional accuracy; thus, it was necessary to regularly check the offset compensation. Secondly, the focusing accuracy was also influenced by the roughness of the machined object. The focusing accuracy could be improved by ensuring the smoothness of the machined object surface; thus, the stainless steel material could be polished before machining to reduce surface roughness.

### 6.3. Comparative Experiment on the Diameter Measurement of Tapered Holes on the Metal Sheet

**Figure 16:**
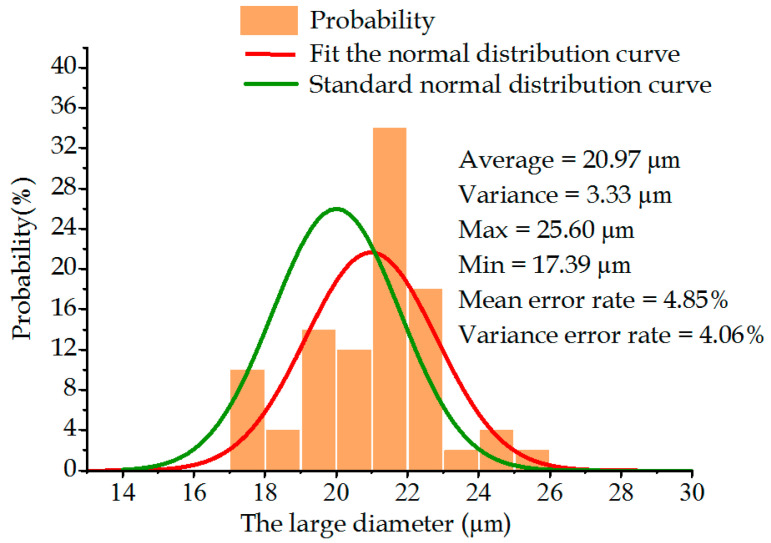
Relationship between the the large diameter and probability of metal sheets.

**Figure 17:**
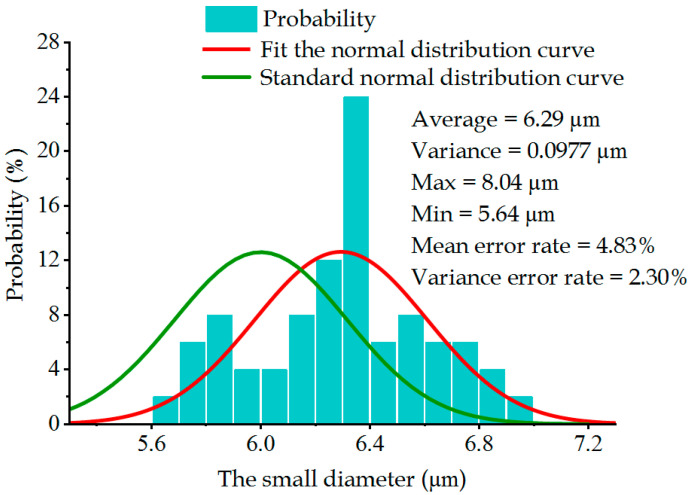
Relationship between the small diameter and probability of metal sheets.

After the error interference in the machining process of the metal sheet was corrected, the laser drilling experiments were performed and compared. Figure 16 and Figure 17 exhibit the relational graphs between the improved large and small diameters of the metal sheet and the frequency. It can be seen that the average value and variance of the small diameter of the metal sheet were 6.29 μm and 0.0977 μm, respectively, and those of the large diameter were 20.97 μm and 3.33 μm, respectively. Compared with laser drilling before the improvement, the average value of the large and small diameters of the metal sheet declined by 2.08 μm and 0.66 μm, respectively, which deviated from the set value by 4.85% and 4.83%, respectively. The variance value of the large and small diameters of the metal sheet declined by 1.87 μm and 0.14 μm, respectively, which deviated from the set value by 4.06% and 2.30%, respectively. These results indicate that the quality of the tapered holes on the metal sheet was improved.

## 7. Conclusions

Tapered holes on the metal sheet, which are important constituents of atomizing devices, exert crucial influences on atomization performance. In order to improve atomization performance, the machining performance of tapered holes was explored, and the main conclusions were drawn as follows:

- Two main factors, external vibration disturbance and internal system errors inside the laser processor, were explored; consequently, the vibration model of the machining device and the laser machining model of three procedures were established, respectively.
- Based on the models and the experimental results, it was found that the errors in diameter caused by these two factors accounted for 20% and 67.87% of the total deviation, respectively.
- An optimization method was proposed, whereby a damping system was added to the machining device, and the diameter of the initial laser spot was corrected. The experimental results showed that the deviation of the average value of the large diameter declined from 15.25% to 4.85%, and the deviation of the small diameter declined from 15.83% to 4.83%.
- The proposed model of laser drilling was established for the machining situation with three processes. The cone angle of tapered holes, the thickness of the metal sheet and the melting point of different materials determined the volume of metal to be machined away, which in turn, determined the number of processes. For other applications, the model of the laser machining must be modified by increasing or decreasing the number of procedures. From the modified model, the diameter of laser spot $d_{1}$ can be obtained, through which the same error reduction results can be obtained.

## Figures and Tables

**Table 1:** Atomizing sheet structure parameter table.

**Parameter**	**Thickness** χ **(mm)**	**Outer Diameter** $a_{1}$ **(mm)**	**Inner Diameter** b **(mm)**		
Piezoelectric ceramics	0.69	16	7.75		
**Parameter**	**Thickness** ε **(mm)**	**Diameter** $a_{2}$ **(mm)**	**Tapered hole number**	**Small diameter** c **(μm)**	**Large diameter** d **(μm)**
Metal sheet	0.05	16	300	6.00	20.00

**Table 2:** Related parameters of the metal sheet.

Parameter (Unit)	Symbol	Value
Large diameter (μm)	$d_{1}$	15.67 *
Small diameter (μm)	$c_{3}$	6.95 **
Pulse energy (J)	E	$2.17\times 10^{-4}$
Thickness (μm)	ε	50
Density ($\mathrm{kg}/m^{3}$)	ρ	8000
Melting temperature (°C)	t	1450
Specific heat capacity (J/(kg·°C))	C	0.45 × $10^{3}$
Latent heat of melting (J/kg)	$L_{m}$	2.47 × $10^{5}$
Latent heat of vaporization (J/kg)	$L_{v}$	6.34 × $10^{6}$

Notes: * represents the experimentally measured result (see details in Section 5.2; ** denotes the experimentally measured result (see details in Section 4).
